# Beyond Ursodeoxycholic Acid: A Comprehensive Review of Second-Line Agents in Primary Biliary Cholangitis

**DOI:** 10.7759/cureus.94172

**Published:** 2025-10-09

**Authors:** Vijay Lakshmanan, Liam Morris

**Affiliations:** 1 Hospital Medicine, Royal Preston Hospital, Lancashire Teaching Hospitals NHS Foundation Trust, Preston, GBR; 2 Gastroenterology, Royal Preston Hospital, Lancashire Teaching Hospitals NHS Foundation Trust, Preston, GBR

**Keywords:** auto-immune, autoimmune liver disease, cholestatic liver disease, elafibranor, obeticholic acid, primary biliary cholangitis (pbc), second line drugs, seladelpar, ursodeoxycholic acid

## Abstract

Primary biliary cholangitis (PBC) is a chronic autoimmune liver disease characterised by progressive bile duct injury and cholestasis. It predominantly affects middle-aged women and typically presents with symptoms such as fatigue, pruritus, and deranged liver enzymes. While ursodeoxycholic acid (UDCA) remains the first-line therapy, a significant proportion of patients fail to achieve adequate biochemical response, leaving them vulnerable to disease progression. Until recently, treatment options for these individuals in the United Kingdom (UK) were limited. However, the therapeutic landscape is evolving with the recent approval of elafibranor and seladelpar, offering new hope for patients and clinicians alike. This review highlights key characteristics of these emerging second-line agents, including their mechanisms of action, administration, safety profiles, and regulatory status in the UK. Special attention is given to the clinical implications of their approval and accessibility within NHS pathways. In addition to disease-modifying therapies, adjunctive strategies for symptom control, particularly for pruritus and fatigue, are also discussed, along with a brief overview of future therapeutic directions. By summarising the expanding treatment arsenal, this review aims to support evidence-informed decision-making and promote timely specialist referral in patients with suboptimal response to UDCA.

## Introduction and background

Primary biliary cholangitis (PBC) is a chronic, immune-mediated liver disease characterised by progressive destruction of the intrahepatic bile ducts [1]. The global burden of PBC has steadily increased, with recent studies estimating that around 18 people per 100,000 have the condition, and approximately 1.8 new cases are diagnosed each year per 100,000 individuals [2]. In the United Kingdom (UK), prevalence is even higher, reaching up to 35 per 100,000, predominantly affecting middle-aged women [1,3]. 

The mainstay of treatment for PBC remains ursodeoxycholic acid (UDCA), a hydrophilic bile acid that improves bile flow and delays disease progression. However, around 40% of patients fail to achieve an adequate biochemical response to UDCA alone, placing them at higher risk of cirrhosis, liver failure, and the need for transplantation [4]. 

Until recently, second-line therapy options for PBC in the UK were limited. The approval of obeticholic acid (OCA) in 2017 marked a significant development. However, its broader use has been constrained by tolerability concerns (most notably dose-dependent pruritus). This adverse effect has previously led to treatment discontinuation in approximately 1-10% of patients, highlighting the need for alternative options in symptomatic individuals [4]. 

In response to the unmet needs among UDCA non-responders, the therapeutic landscape for PBC has recently expanded. Elafibranor, a dual peroxisome proliferator-activated receptors alpha and delta (PPAR-α/δ) agonist, received National Institute for Health and Care Excellence (NICE) UK approval in November 2024 [5], while seladelpar, a selective PPAR-δ agonist, was authorised by the Medicines and Healthcare products Regulatory Agency (MHRA) in January 2025 [6], and is currently under NICE appraisal. 

This review provides an up-to-date overview of these emerging second-line therapies, with particular emphasis on their mechanisms of action, regulatory status within the UK, clinical efficacy, and safety profiles, therefore supporting informed decision-making for clinicians across both general and specialist care settings. In addition, this review outlines adjunctive therapies used for symptom control, particularly with pruritus and fatigue. It concludes with a brief discussion on future directions in PBC treatment, highlighting promising treatments currently in development. 

## Review

PBC is an autoimmune cholestatic liver disease characterised by immune-mediated injury to the small intrahepatic bile ducts, leading to cholestasis, inflammation, and progressive fibrosis. Genetic susceptibility and environmental factors such as smoking or prior infections are thought to contribute to disease initiation. The resulting accumulation of bile acids and subsequent hepatocellular injury form the basis for current therapeutic approaches. Management, therefore, centres around bile-acid modulation, beginning with a first line of UDCA, which improves bile flow and delays disease progression [1]. Patients with an inadequate response or intolerance to UDCA are considered for second-line therapy with obeticholic acid (OCA), which further reduces bile-acid synthesis and cholestatic injury. In those with persistent high-risk disease despite these agents, additional new and repurposed treatments are being evaluated in specialist centres. Together, these therapies aim to interrupt the cycle of cholestasis and fibrosis, slow disease progression, and improve long-term outcomes in patients with PBC [1]. Below, we outline the clinical roles and UK-specific considerations for both established and emerging therapies. 

UDCA 

UDCA remains the cornerstone of first-line treatment for patients with PBC. A naturally occurring hydrophilic bile acid, UDCA acts by replacing the more hepatotoxic natural bile acids, thereby protecting cholangiocytes and hepatocytes from bile acid-induced injury [4]. 

The standard recommended dose is 13-15 mg/kg/day, usually administered in two to three divided doses or as a single oral daily dose. Clinical guidelines support its initiation at diagnosis, regardless of symptom burden or disease stage, as it has been shown to delay histological progression, reduce the risk of liver transplantation, and improve transplant-free survival in responders [4]. 

In addition to promoting bile flow, UDCA provides hepato-protective impacts through several mechanisms. These include reducing inflammation, inhibiting apoptosis (programmed cell death), limiting the development of fibrosis, and modulating immune responses [7]. 

Long-term studies have shown that UDCA significantly improves outcomes, with transplant-free survival rates of around 80% at 10 years. It can reduce the risk of liver transplant or death by more than half, with an estimated 11 patients needing treatment to prevent one such event over five years [7]. UDCA also has a reasonably favourable side effect profile without requiring dose adjustments in patients with renal or liver impairment. Common side effects are mild and may include GI upset, weight gain, or hair thinning [7]. 

Response to UDCA is typically assessed after 12 months of therapy, using biochemical criteria. Several response models exist, but a commonly accepted threshold is a reduction of alkaline phosphatase (ALP) to less than 1.67 times the upper limit of normal (ULN) with normalisation of serum bilirubin levels [4]. ALP is chosen as the principal biochemical marker of response because it closely reflects cholestatic activity and bile duct injury, correlating strongly with disease stage, histological progression, and transplant-free survival. In contrast, bilirubin (although a direct bile metabolite) typically rises only in more advanced disease, when significant ductopenia and fibrosis are already established, making it a less sensitive marker for early response to treatment. The combination of ALP and bilirubin, therefore, provides the most reliable means of assessing therapeutic response and risk stratification in clinical practice [4,7]. Patients who do not meet this response threshold are classified as having an inadequate response, placing them at an increased risk of disease progression and poorer outcomes [4]. 

In clinical practice, up to 40% of patients fail to achieve adequate biochemical response to UDCA monotherapy [7]. These individuals require early identification and consideration for second-line therapy, such as OCA or other second-line agents. Guidelines recommend that response assessment be made at the one-year mark to facilitate timely referral and prevent further disease progression [7]. 

Second-line therapies 

For patients with an inadequate response to UDCA, multiple second-line therapies have become available to mitigate disease progression and improve both biochemical and symptomatic outcomes. These include OCA, elafibranor, and seladelpar, each with distinct mechanisms of action and tolerability profiles. In the following section, we will examine each of these therapies in turn, focussing on their mechanism, clinical evidence, safety profiles, and current role in UK clinical practice. 

OCA

OCA is a semisynthetic bile acid analogue and a potent farnesoid X receptor (FXR) agonist, approved by the NICE in 2017 as a second-line agent for patients with PBC who exhibit an inadequate biochemical response to UDCA or are unable to tolerate it [8]. 

OCA, a synthetic derivative of the naturally occurring bile acid chenodeoxycholic acid, is a potent agonist of FXR [9]. FXR is a nuclear receptor that plays a central role in bile acid homeostasis. FXR activation reduces bile acid production in the liver and enhances its clearance through increased transporter expression. In PBC, where bile acid buildup contributes to liver inflammation and damage, this helps alleviate cholestasis. OCA achieves this by suppressing key enzymes (like CYP7A1) in bile acid synthesis and promoting the removal of bile acids from liver cells into the bile ducts and bloodstream [9]. In addition to its anticholestatic effect, FXR activation has also been shown to reduce liver inflammation and may exert anti-fibrotic effects by modulating inflammatory signaling and sinusoidal cell activity [9]. 

The efficacy of OCA was demonstrated in the Phase 3 POISE trial, which enrolled 216 participants who either had an inadequate response to UDCA or found the side effects of UDCA unacceptable. Participants were randomised to receive either OCA 5 mg (with possible increase to 10 mg), OCA 10 mg, or placebo, in addition to background UDCA therapy in most cases. After 12 months, nearly half of the patients receiving OCA met the primary composite endpoint compared to only 10% in the placebo group (n = 216; 46-47% (OCA) vs 10% (placebo), P < 0.001) [8]. The endpoint was defined as a reduction in alkaline phosphatase (ALP) to below 1.67× the ULN, with at least a 15% decrease in ALP from baseline and a normal serum bilirubin [8]. 

OCA also led to significant reductions in several liver enzymes, including alanine aminotransferase (ALT), aspartate aminotransferase (AST), and gamma-glutamyl transferase (GGT). It additionally reduced conjugated bilirubin and total bile acid levels, reflecting improvement in cholestasis. These biochemical changes were accompanied by decreases in inflammatory markers such as high-sensitivity C-reactive protein (CRP) and tumour necrosis factor-α (TNF-α), consistent with activation of the FXR pathway [8]. However, dose-dependent pruritus was a common side effect, particularly at the 10 mg dose, which led to treatment discontinuation in some cases. Starting at 5 mg and up-titrating if tolerated can help manage this risk [8]. 

Although OCA improves key biochemical markers of disease, such as ALP, bilirubin, and transaminase levels, it has not demonstrated significant effects on non-invasive measures of fibrosis over the study period. In the POISE trial, fibrosis was assessed using transient elastography and the enhanced liver fibrosis (ELF) score. The ELF score incorporates serum components, including hyaluronic acid, procollagen type III N-terminal peptide (P3NP), and tissue inhibitor of metalloproteinase 1 (TIMP-1). Neither transient elastography nor the ELF score showed significant improvement compared with placebo, suggesting that OCA’s therapeutic benefit is primarily biochemical rather than structural [8]. It is also contraindicated in patients with advanced or decompensated liver disease (Child-Pugh B or C) [8,10]. 

Longer-term data from the open-label extension of the POISE trial (long-term safety extension (LTSE)) suggested sustained biochemical benefit at 12 and 21 months of OCA therapy, based on the same composite endpoint and ALP reduction [11]. However, bilirubin results were inconsistent. Moreover, health-related quality of life was assessed using the PBC-40 questionnaire, which showed a numerical worsening in most symptom domains, including fatigue and itch, except for the emotional domain, which remained stable. Pruritus severity also appeared to worsen over time based on patient-reported outcomes using both a visual analogue scale and a pruritus-specific questionnaire. While no deaths were attributed to the drug, nearly all participants experienced some form of adverse event. There were, however, limitations to the study, including its non-randomised design, limited monotherapy data, and a relatively low number of patients completing longer-term follow-up, thereby leaving some uncertainty regarding the long-term safety and efficacy of OCA [11]. 

Elafibranor

Elafibranor is a novel oral agent recently approved as a second-line therapy for patients with PBC who exhibit an inadequate response to UDCA. It functions as a dual agonist of PPAR-α/δ. These are nuclear transcription factors that have a crucial role in regulating bile acid homeostasis, lipid metabolism, inflammation, and fibrogenesis [12]. 

Through PPAR-α activation, elafibranor enhances β-oxidation of fatty acids and promotes bile acid detoxification pathways in hepatocytes. Simultaneously, PPAR-δ activation contributes to anti-inflammatory signalling and modulates immune responses within the liver. Together, these effects reduce cholestasis, improve hepatic biochemistry, and may slow down disease progression [12]. 

The efficacy and safety of elafibranor were demonstrated in the Phase 3 ELATIVE® trial, a multinational, randomised, double-blind study involving 161 patients with PBC who had an inadequate response to or intolerance of UDCA. Participants were randomly assigned in a 2:1 ratio to receive elafibranor 80 mg once daily or placebo, and those already receiving UDCA were allowed to continue it at a stable dose throughout the study. After 52 weeks, 51% of patients treated with elafibranor, whether as monotherapy or in combination with UDCA, achieved the composite primary endpoint, defined as an ALP level below 1.67 times the ULN, with at least a 15% reduction from baseline and normal total bilirubin levels, compared with only 4% in the placebo group (n = 161; 51% (elafibranor) vs 4% (placebo), P < 0.001) [13].

In addition to biochemical improvement, elafibranor was generally well tolerated. In particular, unlike OCA, elafibranor did not exacerbate pruritus, which is a key benefit for patients in whom pruritus is a dominant and debilitating symptom [13]. Improvements in itch-related quality of life were also reflected in scores from patient-reported outcome measures such as the PBC-40 questionnaire and the 5-D itch scale, which showed possible reductions in moderate-to-severe pruritus after 52 weeks of treatment [13]. 

However, a range of adverse effects have been reported with elafibranor. Common side effects include GI symptoms such as abdominal pain, nausea and vomiting, diarrhoea, as well as musculoskeletal complaints like arthralgia. Less frequently, patients may experience weight changes, rashes, or symptoms such as dry mouth and gastroesophageal reflux [14]. The risk of muscle-related toxicity may be increased when used concurrently with statins. Monitoring of symptoms and creatine kinase (CK) levels is therefore advised. Moreover, elafibranor is not recommended during pregnancy due to potential foetal toxicity. Women of childbearing potential should undergo pregnancy testing before commencing treatment and use effective contraception throughout the course of therapy [14]. 

In November 2024, NICE approved elafibranor for use in the UK, marking a major therapeutic development after years of limited second-line options. It is now funded by NHS England and available for prescription in appropriate specialist settings [5]. While long-term outcome data are still being gathered, current evidence supports elafibranor as an effective and safe second-line agent for patients with UDCA-refractory PBC. Its once-daily oral dosing and favourable side effect profile enhance its clinical usability. 

Seladelpar

Seladelpar is another novel agent targeting the PPAR pathway for the treatment of primary biliary cholangitis. While elafibranor is a dual PPAR-α/δ agonist, seladelpar selectively activates the PPAR-δ subtype, offering a more focused approach to modulating bile acid metabolism and hepatic inflammation [15].

PPAR-δ is found in several important liver cell types involved in the progression of PBC, including hepatocytes, cholangiocytes, immune cells (Kupffer cells), and fibrotic cells (hepatic stellate cells) [16-18]. When seladelpar activates PPAR-δ, it stimulates the release of a hormone called fibroblast growth factor 21 (FGF-21), which helps reduce the production of bile acids by lowering the activity of a key enzyme, cholesterol 7α-hydroxylase (CYP7A1), involved in bile acid synthesis [19].

Seladelpar helps reduce liver inflammation by lowering the number of pro-inflammatory macrophages in the liver, supporting a shift of Kupffer cells and other macrophages toward an anti-inflammatory, tissue-healing state [15]. Through this mechanism, seladelpar enhances bile acid regulation, improves lipid handling, and dampens hepatic inflammation [15]. These effects are believed to underlie both its biochemical benefits and its positive impact on pruritus, making it a reasonable alternative to agents such as obeticholic acid, which may worsen cholestatic symptoms. In particular, Seladelpar has been shown to decrease serum interleukin-31 (IL-31) and bile acid levels in patients with PBC [20]. IL-31 is a cytokine associated with the sensation of itch in cholestatic liver disease, and elevated levels have been linked to pruritus severity in PBC. Therefore, reductions in IL-31 and bile acids were closely correlated with improvements in pruritus, suggesting a possible mechanism for seladelpar’s antipruritic effect [20].

In the RESPONSE Phase 3 trial, seladelpar demonstrated strong efficacy and tolerability in patients with PBC. After 12 months of treatment in a group of 193 patients (93.8% of whom received background UDCA therapy), 61.7% of patients receiving seladelpar met the primary biochemical response criteria, compared to just 20% in the placebo group (n = 193; 61.7% (seladelpar) vs 20% (placebo), P < 0.001). Additionally, 25% of patients on seladelpar achieved normalisation of ALP, while none in the placebo group did. Seladelpar also significantly reduced pruritus in patients with moderate-to-severe itch, with a mean improvement of 3.2 points on the pruritus scale by month 6, compared to 1.7 points with placebo [15,21]. 

These results are notably stronger than those seen with OCA in the POISE trial, where approximately 47% of patients achieved the primary endpoint, and pruritus worsened in many [8]. Seladelpar’s efficacy is also favourable compared to elafibranor, which showed around 51% biochemical response rate and modest improvements in itch [13]. Overall, seladelpar appears to offer a potent, well-tolerated option for second-line treatment in PBC, with particular benefit for patients troubled by pruritus [15]. However, as with all recent trials, long-term safety and durability of response require further evaluation through extended follow-up studies.

The drug is administered as a 10 mg once-daily oral tablet and is well tolerated. Common side effects reported were mild and included headache, nausea, and fatigue. Importantly, there were no significant liver-related adverse events or safety concerns raised in the Phase 3 programme [15]. Its favourable safety profile makes it a viable option for patients who are not only biochemically unresponsive to UDCA but also significantly symptomatic. 

While MHRA approval was granted in January 2025, seladelpar is currently undergoing NICE appraisal and is expected to receive NHS approval by late 2025. In the interim, it remains accessible in the UK through specialist hepatology referral pathways [6]. 

**Figure 1:**
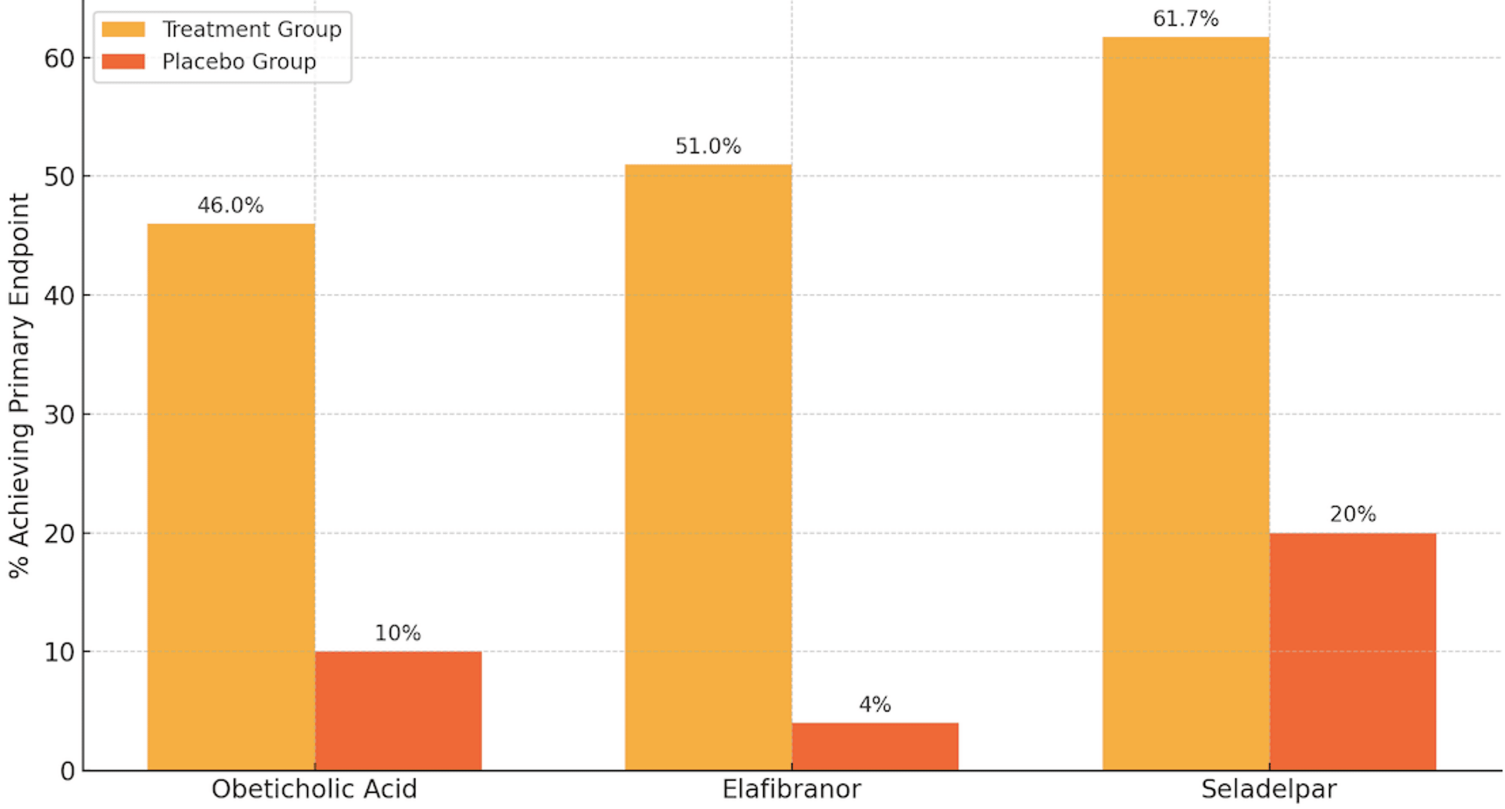
Comparison of primary endpoint achievement in major trials of obeticholic acid, elafibranor, and seladelpar for PBC treatment This bar graph illustrates the percentage of patients achieving the primary composite endpoint in the treatment versus placebo arms of three major Phase 3 trials evaluating second-line therapies for primary biliary cholangitis (PBC). The data are sourced from: (i) POISE Trial for OCA (Nevens et al., 2016 [8]): n = 216; 46–47% (OCA) vs 10% (placebo), P < 0.001; (ii) ELATIVE Trial for elafibranor (Kowdley et al., 2024 [13]): n = 161; 51% (elafibranor) vs 4% (placebo), P < 0.001; (iv) RESPONSE Trial for seladelpar (Hirschfield et al., 2024 [15]): n = 193; 61.7% (seladelpar) vs 20% (placebo), P < 0.001. The graph demonstrates the comparative efficacy of each agent in achieving commonly defined biochemical response criteria among patients with an inadequate response to UDCA. While it provides a useful visual summary, this graph should be interpreted as a rough comparison, as variations exist between trials, particularly in relation to background UDCA therapy duration and inclusion criteria. PBC: primary biliary cholangitis; UDCA: ursodeoxycholic acid; OCA: obeticholic acid

To consolidate the key information discussed above, Table 1 summarises key clinical characteristics of the principal therapies used in the management of PBC [4-6,8,13,15]. This includes their mechanisms of action, route of administration, licensing status, and notable efficacy and safety considerations relevant to UK clinical practice. In addition, Figure 1 presents a comparative bar graph demonstrating the proportion of patients achieving the primary endpoint in the clinical trials of OCA, elafibranor, and seladelpar versus placebo [8,13,15]. 

**Table 1:** Comparison of first-line and second-line therapies for primary biliary cholangitis Data are summarised from pivotal clinical trials referenced in the article, and official regulatory guidance [4-6,8,13,15]. NICE: National Institute for Health and Care Excellence; MHRA: Medicines and Healthcare products Regulatory Agency; PPAR-α/δ: peroxisome proliferator-activated receptors alpha and delta; FXR: farnesoid X receptor

Drug	Mechanism of Action	Dosing	Biochemical Response Rate	Impact on Pruritus	Approval Status (UK)
Ursodeoxycholic Acid (UDCA)	Hydrophilic bile acid; displaces toxic bile salts, improves bile flow	13–15 mg/kg/day	≈60%	No significant effect	First-line standard of care
Obeticholic Acid (OCA)	FXR agonist; reduces bile acid synthesis and inflammation	5–10 mg/day (oral)	≈47% (POISE trial)	May worsen pruritus (dose-dependent)	NICE approved (2017)
Elafibranor	Dual PPAR-α/δ agonist; regulates bile acids, inflammation, and lipid metabolism	80 mg/day (oral)	≈51% (ELATIVE trial)	Neutral effect	NICE approved (2024)
Seladelpar	Selective PPAR-δ agonist; reduces bile acid synthesis and inflammation	10 mg/day (oral)	≈62% (RESPONSE trial)	Improves pruritus	MHRA approved (2025), NICE appraisal pending

Adjunctive therapies for symptom management 

In addition to biochemical progression and risk of liver failure, PBC is marked by symptoms that significantly impair quality of life (most notably pruritus and fatigue). These symptoms often persist despite adequate biochemical control and may require tailored adjunctive therapy. Pruritus, a hallmark of cholestatic liver disease, can affect patients at any stage of PBC, often worsening at night and leading to sleep disturbance and emotional distress [4]. Fatigue, reported in over 50% of patients, is another debilitating complaint that may stem from central, peripheral, or multifactorial origins, often unrelated to the stage of liver disease [22]. 

Effective management of these symptoms is essential in optimising patient well-being and should therefore be considered an integral component of PBC treatment. A stepwise, evidence-based approach to these symptoms has been endorsed by the European Association for the Study of the Liver (EASL) Clinical Practice Guidelines [4]. Below, we outline the commonly used adjunctive therapies based on their mechanism, effectiveness, and safety profiles. 

Management of Pruritus

First-line therapy involves bile acid sequestrants, with cholestyramine being most commonly prescribed. It binds bile salts in the intestine and may reduce systemic pruritogenic load [23]. However, gastrointestinal side effects and drug-binding interactions often limit long-term use. Patients must be counselled to separate cholestyramine from other medications (e.g., UDCA or OCA) by two to four hours to avoid interference with absorption [4]. 

Rifampicin is a second-line agent with good efficacy, acting via activation of the pregnane X receptor, which likely regulates bile acid metabolism and reduces pruritogenic mediators. It also inhibits the activity of the bile salt export pump (BSEP), the key bile acid transporter on the apical membrane of hepatocytes, thereby modulating bile acid transport and synthesis [4]. Caution is required due to potential hepatotoxicity and haemolysis. Monitoring of liver function tests (LFT) is therefore essential during treatment [24]. 

Opiate antagonists, such as naltrexone, can reduce itch intensity by modulating central perception of itch through the endogenous opioid pathway. These agents should be initiated at low doses to prevent withdrawal-like symptoms and monitored for tolerability [25]. Selective serotonin reuptake inhibitors (SSRIs), particularly sertraline, and agents like gabapentin may be used empirically in patients unresponsive to other lines of treatment [1]. While robust trial data are limited, these medications have shown anecdotal benefit in practice and are generally well tolerated [1]. 

A summary of the key agents used in symptom control, including their mechanism, dosing considerations, and limitations, is presented in Table 2 [1,4]. 

**Table 2:** Adjunctive therapies for the management of pruritus in primary biliary cholangitis Data summarised from EASL Clinical Practice Guidelines [4].

Agent	Mechanism of Action	Typical Use	Cautions/Side Effects
Cholestyramine	Binds to bile acids in the gut to reduce pruritogens	First-line for pruritus	GI upset, binds to other drugs. Dose separation needed
Rifampicin	Activates pregnane X receptor to reduce bile acid synthesis	Second-line for pruritus	Hepatotoxicity, haemolysis. LFT monitoring required
Naltrexone	Blocks opioid receptors to modulate itch perception	Third-line for pruritus	Opiate withdrawal symptoms, poor tolerability long term
Sertraline (SSRI)	Modifies central serotonin levels affecting itch processing	Adjunctive therapy for resistant pruritus	Dry mouth, somnolence, limited trial data
Gabapentin	Modulates neuronal excitability; increases itch threshold	Adjunctive therapy for resistant pruritus	Dizziness, fatigue, evidence limited

Management of Fatigue

Fatigue in PBC is complex and often multifactorial. While not correlated with disease stage or biochemical activity, it is a major contributor to poor health-related quality of life. No pharmacological therapy has demonstrated clear efficacy in treating fatigue. Therefore, management focuses on identifying and addressing contributing factors such as hypothyroidism, anaemia, sleep disturbance, and concurrent medications (For example, beta-blockers) [4]. 

Structured patient support, coping strategies to combat social isolation, and use of validated tools such as the PBC-40 quality of life questionnaire are encouraged [26]. Although experimental, structured exercise and psychological support may yield modest benefit [4]. 

Future directions

The therapeutic landscape of PBC is rapidly evolving, with multiple agents poised to transform both disease modification and symptom control. While recent regulatory approvals of elafibranor and seladelpar have introduced novel PPAR agonists with favourable safety and efficacy profiles, future therapeutic strategies must focus on improving long-term biochemical response rates, enhancing transplant-free survival, and developing treatments that directly target symptoms refractory to existing therapy.

Beyond pruritus, which remains one of the most common and disabling complaints, other factors such as fatigue, sicca complex (dry eyes and mouth), sleep disturbance, autonomic dysfunction, and mood disorders significantly impair quality of life. Persistent symptoms like itch, cognitive dysfunction, and social isolation continue to represent key unmet needs in PBC management [4]. Future drug development should therefore aim not only to halt disease progression but also to meaningfully improve the multidimensional quality-of-life burden associated with this chronic autoimmune condition.

Building on the success of PPAR-targeted therapies, several other investigational agents are in development, aiming to further optimise biochemical response and address symptom domains inadequately managed by current treatments.

Saroglitazar, a dual PPAR-α/γ (alpha/gamma) agonist, showed promising results in a phase 2 trial, with nearly 50% reduction in ALP and 71% of patients achieving the composite endpoint at the 4 mg dose. However, elevations in liver enzymes prompted discontinuation in some cases. Phase 3 trials are therefore evaluating lower, potentially safer dosing strategies [27,28]. 

Setanaxib is a selective NOX (NADPH oxidase) 1/4 enzyme inhibitor that targets oxidative stress, an important driver of hepatocellular injury and fibrosis in PBC. In PBC, chronic inflammation activates hepatic stellate cells and NOX enzymes, leading to the generation of reactive oxygen species that contribute to bile duct damage, inflammation, and fibrotic progression. By inhibiting NOX1/4, setanaxib reduces oxidative injury and may slow disease progression. In a phase 2 trial, setanaxib (400 mg once or twice daily) administered with UDCA led to modest reductions in GGT and ALP, with the greatest improvement observed in patients with higher baseline liver stiffness. Post-hoc analysis also suggested a potential improvement in fatigue symptoms [29].

Symptom control, particularly with pruritus, remains a key area of unmet need. While cholestyramine, rifampicin, and naltrexone form the backbone of current adjunctive therapy, their use is often limited by poor tolerability, drug-to-drug interactions, and variable efficacy. Newer targeted agents are being developed to overcome these challenges by acting on specific molecular pathways involved in cholestatic itch. Emerging treatments for pruritus in PBC include bile acid transport inhibitors, which reduce systemic bile acid accumulation, and drugs acting on neural itch pathways, such as kappa-opioid receptor agonists and MRGPRX4 antagonists, offering the potential for greater efficacy and fewer systemic side effects [27].

Linerixibat, an apical sodium-dependent bile acid transporter (ASBT), also known as ileal bile acid transporter (iBAT) or SLC10A2 inhibitor, has shown promise in reducing itch severity and improving quality of life at specific doses in a phase 2 trial, although primary endpoints were not met across all groups [27,30]. Other new therapies under investigation include other iBAT/ASBT inhibitors (e.g., volixibat), kappa-opioid receptor agonists (e.g., difelikefalin), and agents targeting the MRGPRX4 receptor (e.g., EP547), reflecting a growing focus on addressing refractory cholestatic pruritus [27]. 

Together, these emerging therapies reflect a growing shift toward personalised, symptom-targeted treatment in PBC. As the treatment landscape evolves, integration of novel agents will be key to improving long-term outcomes, thereby paving the way into the next chapter of disease management.

## Conclusions

This review highlights the evolving management of PBC, with a focus on second-line therapies including OCA, elafibranor, and seladelpar. These agents offer alternative treatment options for patients with an inadequate response to first-line therapy. Adjunctive therapies for managing pruritus and fatigue are also reviewed, underscoring the need for a holistic approach to care. By exploring both current treatments and future therapeutic directions, this report emphasises the importance of early intervention and personalised management in improving patient outcomes. 
